# Graphene and graphene oxide with anticancer applications: Challenges and future perspectives

**DOI:** 10.1002/mco2.118

**Published:** 2022-02-09

**Authors:** Ali Shafiee, Siavash Iravani, Rajender S. Varma

**Affiliations:** ^1^ Department of Chemistry Cape Breton University Sydney Canada; ^2^ Faculty of Pharmacy and Pharmaceutical Sciences Isfahan University of Medical Sciences Isfahan Iran; ^3^ Regional Centre of Advanced Technologies and Materials Czech Advanced Technology and Research Institute Palacky University in Olomouc Olomouc Czech Republic

**Keywords:** cancer nanotherapy, graphene oxide, graphene‐based nanomaterials, graphene

## Abstract

Graphene‐based materials have shown immense pertinence for sensing/imaging, gene/drug delivery, cancer therapy/diagnosis, and tissue engineering/regenerative medicine. Indeed, the large surface area, ease of functionalization, high drug loading capacity, and reactive oxygen species induction potentials have rendered graphene‐ (G‐) and graphene oxide (GO)‐based (nano)structures promising candidates for cancer therapy applications. Various techniques namely liquid‐phase exfoliation, Hummer's method, chemical vapor deposition, chemically reduced GO, mechanical cleavage of graphite, arc discharge of graphite, and thermal fusion have been deployed for the production of G‐based materials. Additionally, important criteria such as biocompatibility, bio‐toxicity, dispersibility, immunological compatibility, and inflammatory reactions of G‐based structures need to be systematically assessed for additional clinical and biomedical appliances. Furthermore, surface properties (e.g., lateral dimension, charge, corona influence, surface structure, and oxygen content), concentration, detection strategies, and cell types are vital for anticancer activities of these structures. Notably, the efficient accumulation of anticancer drugs in tumor targets/tissues, controlled cellular uptake properties, tumor‐targeted drug release behavior, and selective toxicity toward the cells are crucial criteria that need to be met for developing future anticancer G‐based nanosystems. Herein, important challenges and future perspectives of cancer therapy using G‐ and GO‐based nanosystems have been highlighted, and the recent advancements are deliberated.

## Graphical abstract

Important challenges, recent advancements, and future perspectives for deploying graphene‐ and graphene oxide‐based nanosystems in cancer therapy are deliberated. Notably, large surface area, ease of functionalization, high drug loading capacity, and reactive oxygen species induction potentials have rendered these structures promising candidates for cancer therapy applications. The ease of functionalization bodes well for their utility in treating assorted types of cancers.

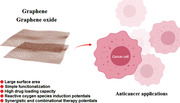

## 1 INTRODUCTION

Graphene (G), the two‐dimensional and hexagonally bonded sp^2^ hybridized carbon structure with extraordinary characteristics, has garnered huge interdisciplinary attention in different fields of science and engineering over the last half‐century. The single, multi‐layered (less than 10) and flatted honeycomb structure of G possesses unique attributes namely high hardness, resistance, thermal and electrical conductivity, optical transmittance, infinite surface area, among others.^1^, ^2^, ^3^, ^4^, ^5^ Graphene oxide (GO) is the oxidized version of G, which is usually produced under harsh oxidation conditions. GO possesses numerous oxygen‐bearing functionalities, such as hydroxyl, carboxylic, and epoxide groups on the carbon surface, rendering it more hydrophilic than G.^6^, ^7^, ^8^, ^9^ The incorporation of G layers in nanocomposites is one of the methods for controlling and improving their area of surface, mechanical/electrical attributes, and thermal conductivity.^10^, ^11^, ^12^ These G‐ and GO‐based nanocomposites with high surface area, ease of functionalization, high drug loading capacity, and reactive oxygen species (ROS) induction potential are promising candidates aimed for the targeted transport of anticancer drugs/genes and diagnostic agents.^13^, ^14^, ^15^, ^16^ Assorted G‐ and GO‐based derivatives in association with multifunctionalization processes can help assist in improving the optical and electrical properties as well as poor solubility in aqueous solutions.^17^, ^18^, ^19^, ^20^ These nanosystems with better biodistribution of drugs, low adverse effects on healthy cells, high selectivity/sensitivity, and higher local therapeutic absorption have garnered a lot of attention.^21^ However, complex synthetic procedures, potential inflammatory effects, possible accumulation in the spleen, likely immunogenicity, cell disruption in higher concentration, and the need for comprehensive in vivo studies/protein folding studies, are their important limitations.^22^, ^23^

G and GO and their corresponding nanocomposites have shown promising applicability for biosensing/bioimaging,^24^ nano‐detecting/labeling,^25^ gene/drug delivery,^26^ and tissue engineering/regenerative medicine,^27^ among others. Nanocarriers comprising G or GO have been deployed for the delivery of anticancer agents with high selectivity/specificity and drug loading capacity.^28^, ^29^ The G‐based advanced functional structures with large surface areas, ease of functionalization/modification, and photothermal features are attractive for cancer nanotherapy.^30^, ^31^ For instance, reduced‐GO structures with good biocompatibility have been fabricated using *Euphorbia heterophylla*, and their cytotoxicity evaluated against A549 and HepG2 human cancerous cells; high cytotoxic effects were observed in vitro, but further studies are warranted to analyze their other biomedical potentials.^32^ Additionally, reduced‐GO materials with dose‐dependent cytotoxicity effects against MCF‐7 cells have been generated by applying *Bacillus marisflavi* as the stabilizer and reductant agent. These bacterially reduced‐GO materials (∼60 μg ml^–1^) could increase the formation of ROS and initiate the release of lactate dehydrogenase.^33^ Han et al.^34^ have reviewed the functionalization and optimization strategies of GO‐centered nanomaterials for drug/gene transport. Various strategies including non‐covalent and covalent (e.g., addition, condensation, and nucleophilic/electrophilic substitution) have been widely explored for the functionalization of G and GO. Increased electrical conductivity, enhanced dispersibility, improved functionality, and good biocompatibility have been reported as outstanding benefits of these functionalized materials. However, some of these functionalization techniques such as addition may suffer from difficulty in controlling, thus controllable selective strategies should be further explored by researchers.^35^, ^36^

For constructing advanced G‐based nanosystems for diagnosis and treatment of cancers, several challenging issues should be considered such as flexibility, biocompatibility, biodegradability, toxicity, surface functionizability, and fluorescence quenching potentials.^37^, ^38^, ^39^, ^40^, ^41^, ^42^ The surface modification and functionalization of G‐based materials can be performed by different polymeric materials.^20^, ^43^ Bioactive materials (e.g.*, L*‐ascorbic acid, chitosan, and gelatin) can be deployed for surface functionalization of these G‐based materials for improving biocompatibility and targeting features. The surface modification of these materials has been reported by introducing a variety of functional groups, helping to adjust and manipulate their surfaces and improve their properties and activities in the form of hybrid materials; galactose, hyaluronic acid, and folic acid are some important compounds reported for improving the targeting and selectivity of anticancer delivery systems.^44^ Herein, important challenges and future perspectives of G‐ and GO‐based materials for cancer therapy are highlighted, with deliberations on recent advances.

## 2 PREPARATION TECHNIQUES

The prodigious intrinsic properties of G and GO make them a high demand material for deployment in diverse research areas such as water treatment,^45^, ^46^, ^47^ air purification,^48^, ^49^, ^50^, ^51^ bactericidal,^52^ cell imaging,^53^, ^54^ medical and life science,^50^, ^55^, ^56^, ^57^, ^58^, ^59^ drug delivery,^60^, ^61^ tissue engineering,^62^, ^63^ energy‐related researches,^64^, ^65^, ^66^, ^67^, ^68^, ^69^ among others. In spite of the fact that G's and GO's potential has been promisingly ascertained; their preparation is relatively difficult and expensive that may restrict their utilization on large industrial scales.^70^ Four basic methods have been established for oxidizing the G to produce GO, comprising Staudenmaier,^71^ Hoffmann,^72^ Brodie,^73^ and Hummers processes,^74^ and modification thereof to render them more efficient, cheaper, and environmentally friendlier, but the main challenges still exist.^75^, ^76^, ^77^ Although other techniques such as chemical exfoliation^78^, ^79^ and chemical vapor deposition^80^ have been developed for the synthesis of G and GO, these methods are expensive and require specialized instrumentation. Additionally, the generation of NO_2_, N_2_O_4_, ClO_2,_ and other toxic or explosive gases during these methods is another drawback that must be considered seriously from an environmental viewpoint.^81^ As the up‐scalable preparation of G‐based structures necessitates expensive materials, complex instruments, and sometimes is ecologically unfriendly; thus, there is an urgent demand for synthesizing these materials via simple and eco‐friendly methods. The sustainable production of these G‐based materials by applying agricultural wastes (e.g., walnut shells and husk) is one of them. Besides, the requirement of high temperature and production of some toxic syngas may cause some environmental problems that need to be addressed in future studies to make the final product more sustainable.

## 3 G‐ AND GO‐BASED NANOSYSTEMS FOR CANCER THERAPY

### 3.1 Photothermal therapy

**FIGURE 1:**
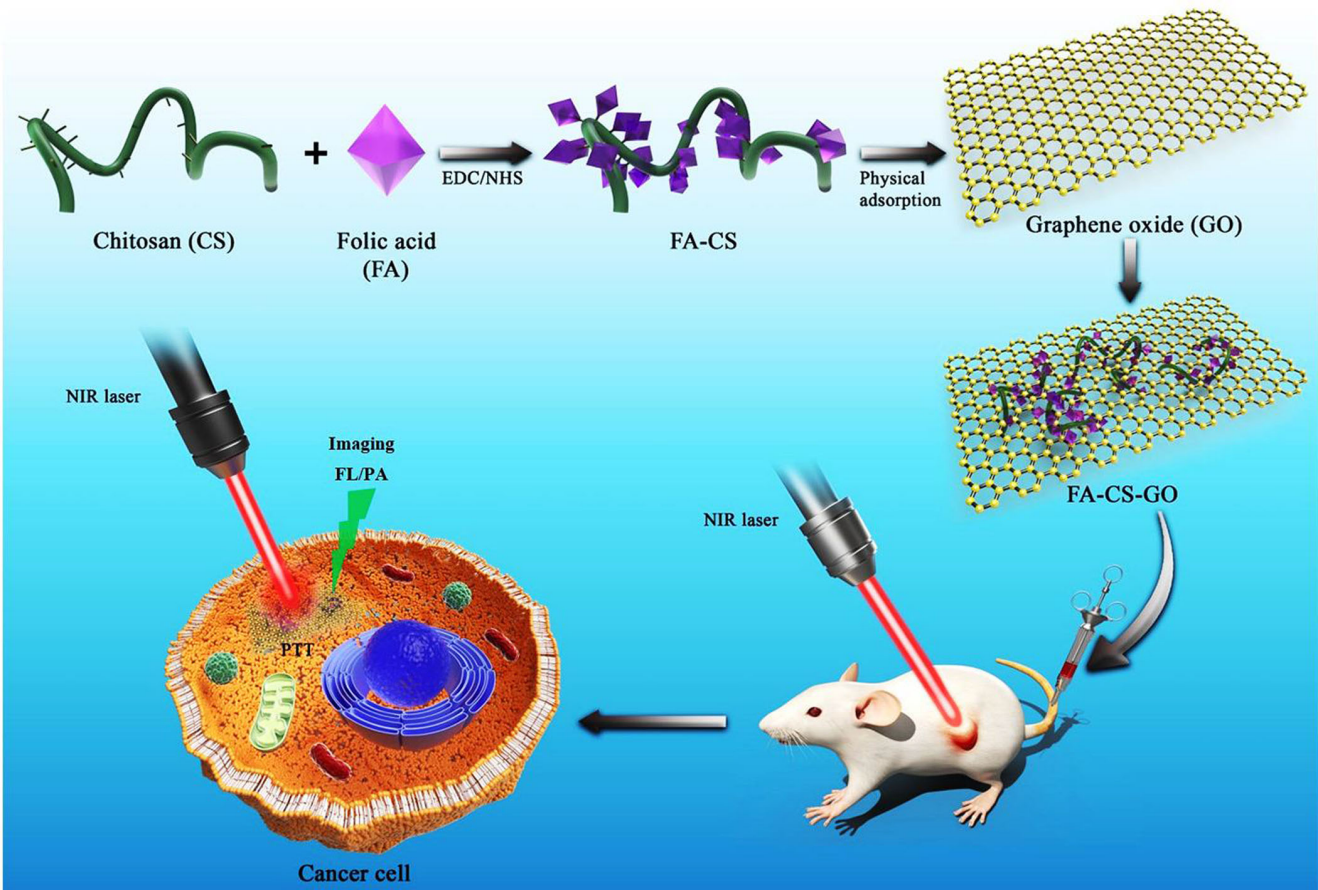
The chitosan (CS)‐functionalized graphene oxide (GO) nanosheets were conjugated with folic acid (FA) for targeted photothermal tumor therapy guided by photoacoustic imaging. Reproduced with permission from Elsevier, 2020^84^

Cancer is often a fatal disease that results in deaths worldwide, thus detection in primary stages and effective treatment strategies are very essential for improving the rate of survival in patients with cancers.^82^ G‐based materials with their unique physicochemical properties can be employed for the detection and treatment of cancers. For instance, when GO was combined with polyethylene glycol (PEG), it exhibited photothermal therapy effects against cancers and tumors via the induction of heating effect in macrophages, in vitro and in vivo.^83^ The macrophage cell lines RAW264.7 were treated with near‐infrared (NIR) light irradiation, and their polarization status was evaluated by flow cytometric and mRNA expression study. GO‐PEG had high thermal stability, improved biocompatibility, and significant photothermal influence. Notably, these photothermal structures alleviated interleukin‐4‐induced M2 polarization of macrophages and regulated their antitumor potentials. Thus, human osteosarcoma lost their migration and invasion potentials, instigating suitable antitumor effects.^83^ Additionally, chitosan‐functionalized GO nanoplatforms were conjugated with folic acid with the purpose of photothermal cancer therapy guided by NIR fluorescence and photoacoustic imaging; the cancerous cells were completely destroyed under laser irradiation, in vitro.^84^ Also, in vivo studies indicated that the tumors were totally obstructed with no recurrence within 20 days, after the deployment of this targeted nanosystem under laser irradiation (Figure 1).^84^

### 3.2 Delivery of anticancer agents

G‐based nanostructures incorporated with anticancer drugs were constructed from GO, G quantum dots (GQDs), and curcumin, with high stability and effective delivery of curcumin inside the cancerous cells.^85^ The complexes, GO‐curcumin and GQDs‐curcumin were evaluated in various ratios against human breast cancer cell lines MDA‐MB‐468 and MCF‐7. As a result, cell viability of more than 75% could be detected from these samples after 48 h of incubation with the cell lines, whereas, by applying curcumin alone (about 100 μg ml^–1^), the cell viability was ∼40%. The corresponding cell death results were ∼60, 80, and 95% at 100 μg ml^–1^ after 48 h of the treatment process, respectively.^85^ In addition, silver‐GO nanocomposites (∼20‐100 μg ml^–1^) have been studied against cancerous cells,^86^ where they displayed suitable cytotoxic effects, but their efficiency was lower than free silver (Ag) nanoparticles with smaller size and better uptake.^87^

Cu_2_O nanoparticles (∼4 nm) were decorated on GO for efficient and selective cancer nanotherapy.^87^ The anticancer effects of these Cu_2_O‐GO nanocomposites were studied against HK‐2, 231, and A549 cells in vitro beneath the visible light irradiation.^87^ Besides, the formulated GO‐PEGylated folate nanocarrier was examined in acidic (pH = 5.0) and physiological (pH = 7.4) environments.^88^ Consequently, ∼21.5% and 71.0% of loaded camptothecin anticancer drug could be released under physiological and acidic conditions, after 48 h of treatment, respectively. The images of confocal microscopy obtained from the treated HeLa cells (after 8 h) by the GO‐PEGylated folate nanosystem illustrated the blue and green fluorescence emission of camptothecin from the nucleus and cytoplasm, respectively, demonstrating targeted drug delivery to the cells.^88^ Zhou et al.^89^ introduced a smart multifunctional MnO_2_‐doped GO nanosystem for the delivery of cisplatin and photosensitizer (Ce6). Consequently, the decomposition of hydrogen peroxide into oxygen was catalyzed to ease the hypoxia of the tumor, and the level of glutathione was reduced in targeted tumors; Mn^2+^ was continuously generated for progressing Fenton‐like reaction, thus providing improved antitumor effects. Notably, hyaluronic acid was applied for modifying the surface of the prepared nanosystem to improve its targeting properties, causing increased cellular toxicity and growth inhibition of tumors.^89^

Folic acid has been decorated on the GO‐based nanocomposite functionalized with PEG for delivering paclitaxel.^90^ The cellular toxicity analysis of this nanosystem demonstrated good biocompatibility and low cytotoxicity; the cell viability being ∼60% and 30% after treating by free paclitaxel drug and the designed nanosystem, respectively. The images of fluorescence microscopy evaluations from the nanosystem proved paclitaxel delivery into the targeted tumor cells with high efficiency. Consequently, by increasing the concentration of a drug, the blue fluorescence emission was also decreased, indorsing the reduction in cell number and the successful entrance of drug nanocarrier into the cells.^90^ In another study, a nanosystem was developed for targeted delivery of doxorubicin, based on the strong conjunction between acidic functional groups of tumors and the hydroxyl groups of G,^91^ when PEG was added for enhancing the biocompatibility of hydroxylated G fabricated via the solid‐state ball milling technique. The cell viability of tumor cells (OCM‐1) and normal cells (ARPE‐19) were less than 10% and 80%, respectively by treating with the nanosystem (10 μg ml^–1^) after 48 h. The confocal microscopy analysis revealed that the hydroxylated GO could be detected inside the cells after 12 h; however, the hydroxylated GO could be found around the cells after 48 h, and then it disappeared after 60 h. Results of this study illustrated that the nanocomposite had suitable antitumor effects against OCM‐1 tumors and exhibited low toxicity to the normal cells.^91^

A GO‐based nanosystem was developed for targeted fluorouracil (FU) delivery.^92^ The cell viability evaluations demonstrated no noticeable toxicity at various concentrations; therefore, further in vivo analysis should be conducted on the prepared nanosystem, especially for anticancer drug delivery. It was indicated that by loading FU on this nanosystem, the cellular viability was increased as nanocarrier could reduce the toxic influence of FU on normal tissues thus improving the biocompatibility.^92^ Besides, an innovative nanocarrier with controllable release features was developed for delivering chlorambucil anticancer drug,^93^ which was prepared using gelatin and reduced‐GO functionalized with folic acid. In vitro drug release was analyzed in 3 mediums including phosphate buffer solution as simulated blood (pH = 7.4), colonic fluid (pH = 5.4), and gastric fluid (pH = 1.2) by applying varying reduced‐GO concentrations; higher release rates could be detected under acidic conditions compared to the neutral conditions. The cell viability evaluations revealed that the prepared nanocomposite had low cytotoxicity; the results from cellular viability analysis (500 μg ml^–1^) were ∼11.7% and 28% for free chlorambucil and chlorambucil‐loaded nanocomposite, respectively.^93^

The phospholipid‐based amphiphilic polymer was deployed for modifying the reduced‐GO to improve the transfection of small interfering RNA (siRNA).^94^ The prepared nanocarrier could deliver siRNA without enzymatic degradation when compared to the free siRNA.^94^ Additionally, the modified GO could be employed as a nanocarrier for transferring siRNA into cells.^95^ After formulating the GO/poly‐*L*‐lysine hydrobromide/folic acid and GO/poly‐*L*‐lysine hydrobromide platforms, doxorubicin and siRNA were loaded on them and the corresponding delivery and cytotoxicity issues were analyzed on HeLa cells; no significant toxic effects could be detected on HeLa cells even at a high concentration of G‐based nanosystem (∼120 μg ml^–1^). The tumor growth was inhibited in the presence of the siRNA gene, and the nanocarrier for siRNA had positive effects while free siRNA demonstrated no noticeable gene silencing effect. Therefore, this nanosystem can be suggested for delivering siRNA genes and silencing the specified gene expression.^95^

Doxorubicin has been loaded onto GO hybridized nanogels, which were employed for photothermal therapy by NIR laser irradiation (wavelength of 808 nm).^96^ As a consequence, the transport of anticancer drugs into A549 cells was improved by applying nanogels. Remarkably, the toxicity effects of these prepared nanogels against the A549 cancer cells were improved by laser treatment, due to the thermal absorption of GO under laser irradiation.^96^ Besides, the G hydrogels functionalized with branched polyethyleneimine were explored for delivering doxorubicin with good biocompatibility and photothermal therapy of breast cells. A combination of chemotherapy with photothermal therapy reduced the cancer cells to ∼33 % while the utilization of doxorubicin‐loaded G hydrogels without laser irradiation decreased the breast cancer cells to ∼66.7%.^97^

Magnetic GO nanostructures were coated by poly lactic‐co‐glycolic acid for the delivery of 5‐iodo‐2‐deoxyuridine to stimulate radio‐sensitizing influences on patients with glioblastoma.^98^ Analytical studies showed that suitable magnetic targeting and improved penetration through the blood‐brain barrier could be obtained. Also, the synergistic outcome was reported by applying this nanocarrier, providing the effective inhibition and apoptosis against C6 glioma tumor, extended circulation half‐life (more than 140 h), increased dose enhancement factor, and enhanced radio‐sensitizing effects.^98^ Additionally, gold nanorods were loaded on GO nanocomposites using polydopamine for targeted doxorubicin delivery to the cancerous cells. These nanosheets had low cytotoxicity and significant biocompatibility even at a 250 μg ml^–1^ concentration after 48 h of the treatment.^99^

**FIGURE 2:**
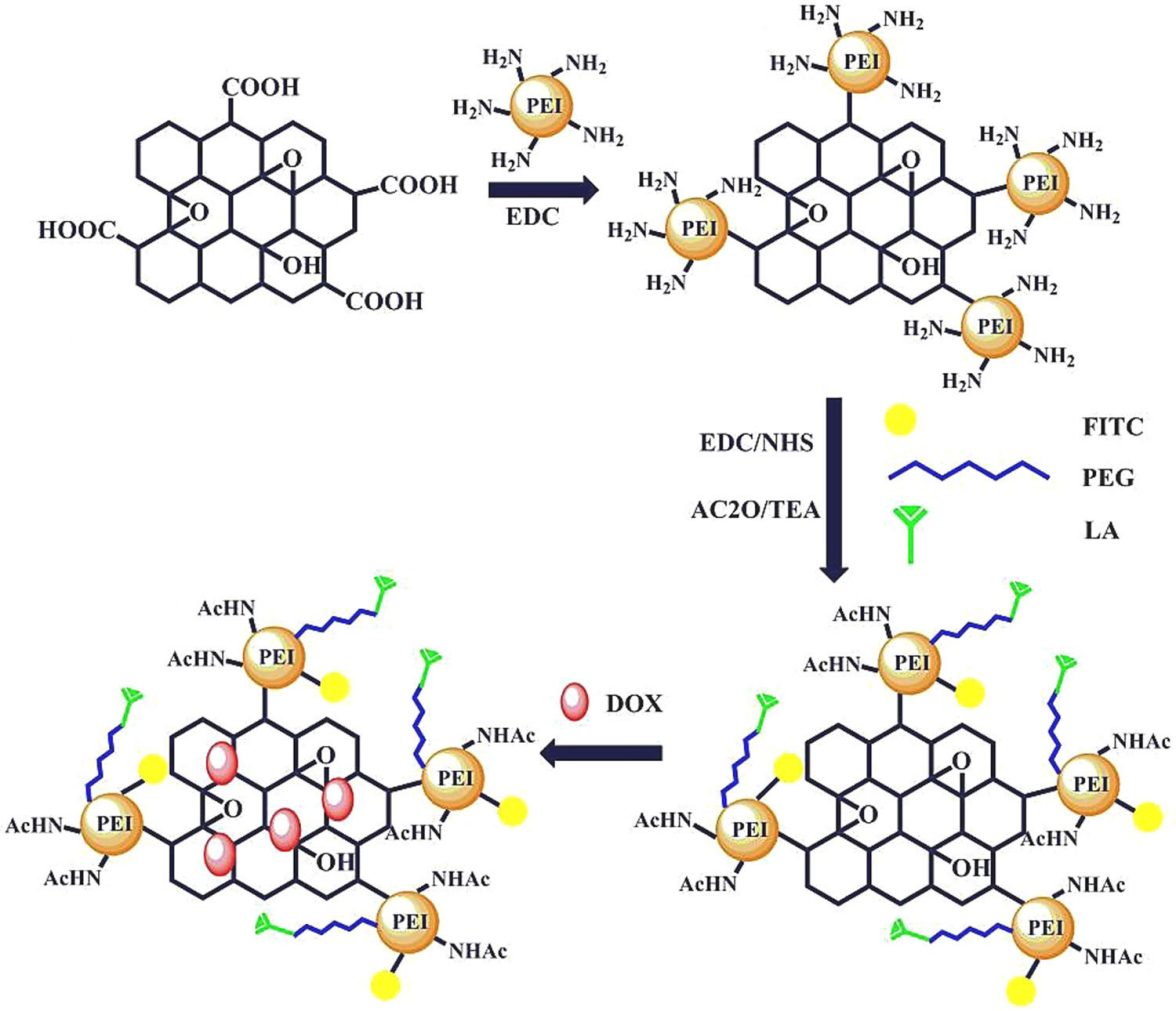
Preparative process of multifunctional graphene oxide (GO)‐based structures with pH‐sensitive and controllable drug delivery properties. Reproduced with permission from Elsevier, 2016^100^

**FIGURE 3:**
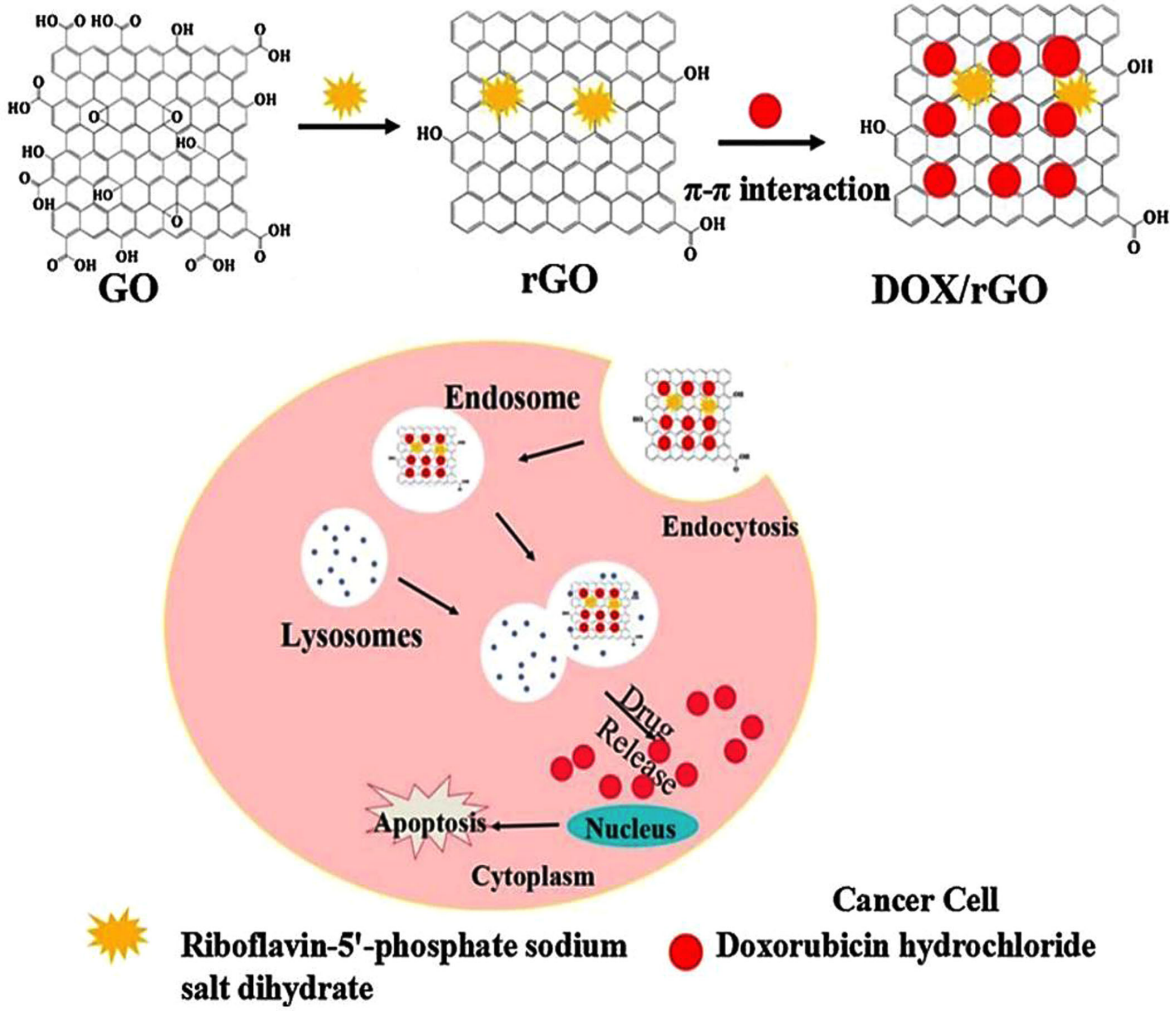
Reduced graphene oxide (GO) for doxorubicin anticancer delivery with pH‐dependent behavior. rGO: reduced GO, DOX: doxorubicin. Reproduced with permission from Elsevier, 2015^101^

Multifunctionality is one of the important criteria for controlling and treating cancers, as it was indicated that the multifunctionalized GO‐based platforms had efficient doxorubicin drug delivery as well as inhibitory effects against hepatocarcinoma cancerous cells. The surface functionalization was performed by deploying polyethyleneimine (PEI) modified with PEG‐linked lactobionic acid and fluorescein isothiocyanate, followed by acetylation of the residual amine groups from PEI.^100^ Among the important properties of this nanocarrier has been suitable cell feasibility in the examined strength span, thus the nanosystem demonstrated improved target specificity and pH‐sensitive release behavior with high growth inhibition effects to the cancerous cells (Figure 2).^100^ Reduced GO nanocarriers were fabricated for pH‐sensitive doxorubicin drug delivery. The prepared nanosystem demonstrated suitable safety/stability profile and high drug loading capacity with pH‐sensitive and sustainable/controllable release behavior. This nanohybrid system illustrated cytotoxicity activity to MCF‐7 and A549 cells via a nonspecific endocytosis mechanism (Figure 3).^101^ It was revealed that the conjugation of GO‐based nanoplatforms with zoledronic acid could lead to producing nanosystems with optimum performance against breast cancer, providing synergistic effects for treating osteoporosis and metastasis.^102^

Functionalized GO‐based nanocomposites have been designed with the purpose of anticancer drug delivery. For instance, a nanocarrier with good biocompatibility and biodegradability features was constructed using gelatin and reduced GO nanosheets functionalized with folic acid for the delivery of chlorambucil anticancer drug. This nanosystem showed controlled release behavior with significant loading capacity. Consequently, the drug release rate was higher under acidic conditions in comparison with the neutral environments.^93^ Additionally, the non‐covalent functionalized GO by Pluronic F127 molecules was introduced for tumor‐targeting therapy. Doxorubicin was loaded onto the prepared nanosystem with high loading capacity and efficiency (∼83%) could induce a higher apoptosis rate (∼12.27 %) of U251 cells compared with free doxorubicin (∼8.20 %).^103^ Hamblin and co‐workers^104^ have designed GO‐based polymeric nanocomposites (∼51 nm) for the delivery of doxorubicin against breast cancer; the drug release being 24.7% and 41.2% under neutral and acidic environments after 72 h.^104^ To develop a dual‐drug loaded nanosystem for combinational cancer therapy, cisplatin and doxorubicin were loaded into a nanoplatform constructed from GO and PEG.^105^ Consequently, the designed nanosystems could be efficiently delivered into tumor cells, introducing noticeable cell apoptosis and necrosis; these agents could inhibit the growth of tumor cells with enhanced efficacy, the rate of promoted apoptosis and necrosis effects on cancerous cells being ∼18.6%.^105^

### 3.3 Combinational cancer therapy

#### 3.3.1 Radiotherapy and photothermal therapy

Combination therapy with lower toxicity and improved targeting benefits through functionalized nanostructures has been one of the topics of interest for scientists in the field of cancer treatment. It was indicated that Fe_3_O_4_@Au/reduced GO nanomaterials could be designed via hydrothermal reaction for combinational therapy via both radiotherapy and photothermal therapy approaches. Accordingly, the efficiency of photothermal conversion was about 61%. These nanosystems showed good biocompatibility with suitable cytotoxicity against oral squamous carcinoma KB cell lines.^106^

#### 3.3.2 Chemo‐photothermal and chemo‐photodynamic therapy

**FIGURE 4:**
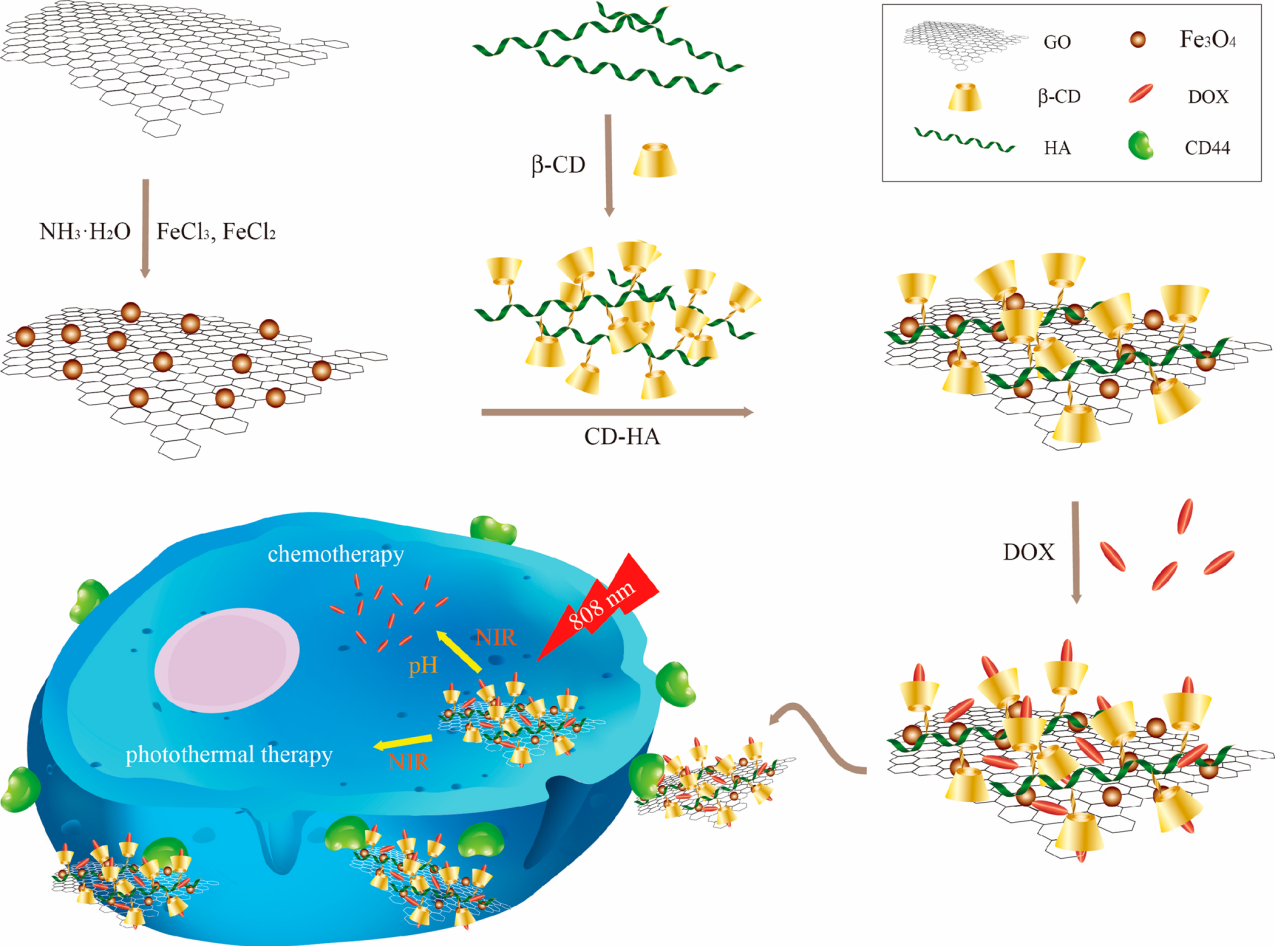
The polymers of *β*‐cyclodextrin (*β*‐CD)‐hyaluronic acid (HA) were attached to Fe_3_O_4_‐GO nanocomposites for targeted chemo‐photothermal therapy of tumors cells. DOX: doxorubicin. Reproduced with permission from MDPI (CC BY 4.0)^107^

A nanoplatform was developed via the attachment to Fe_3_O_4_‐GO polymers emanating from *β*‐cyclodextrin‐hyaluronic acid;^107^ it could be simply separated magnetically and demonstrated significant biocompatibility, suitable dispersibility in water, and photothermal heating via high NIR. The application of hyaluronic acid‐*β*‐cyclodextrin combination could increase the drug (doxorubicin) packing capacity to more than 485.43 mg g^–1^. Notably, the prepared nanosystem provided a rapid photothermal reaction to perform the NIR‐stimulated release of anticancer drugs in solvents with low acidity. The doxorubicin‐loaded nanocomposites revealed CD44 receptor facilitated active‐directing identification together with chemo‐photothermal synergistic antitumor effects (Figure 4).^107^

**FIGURE 5:**
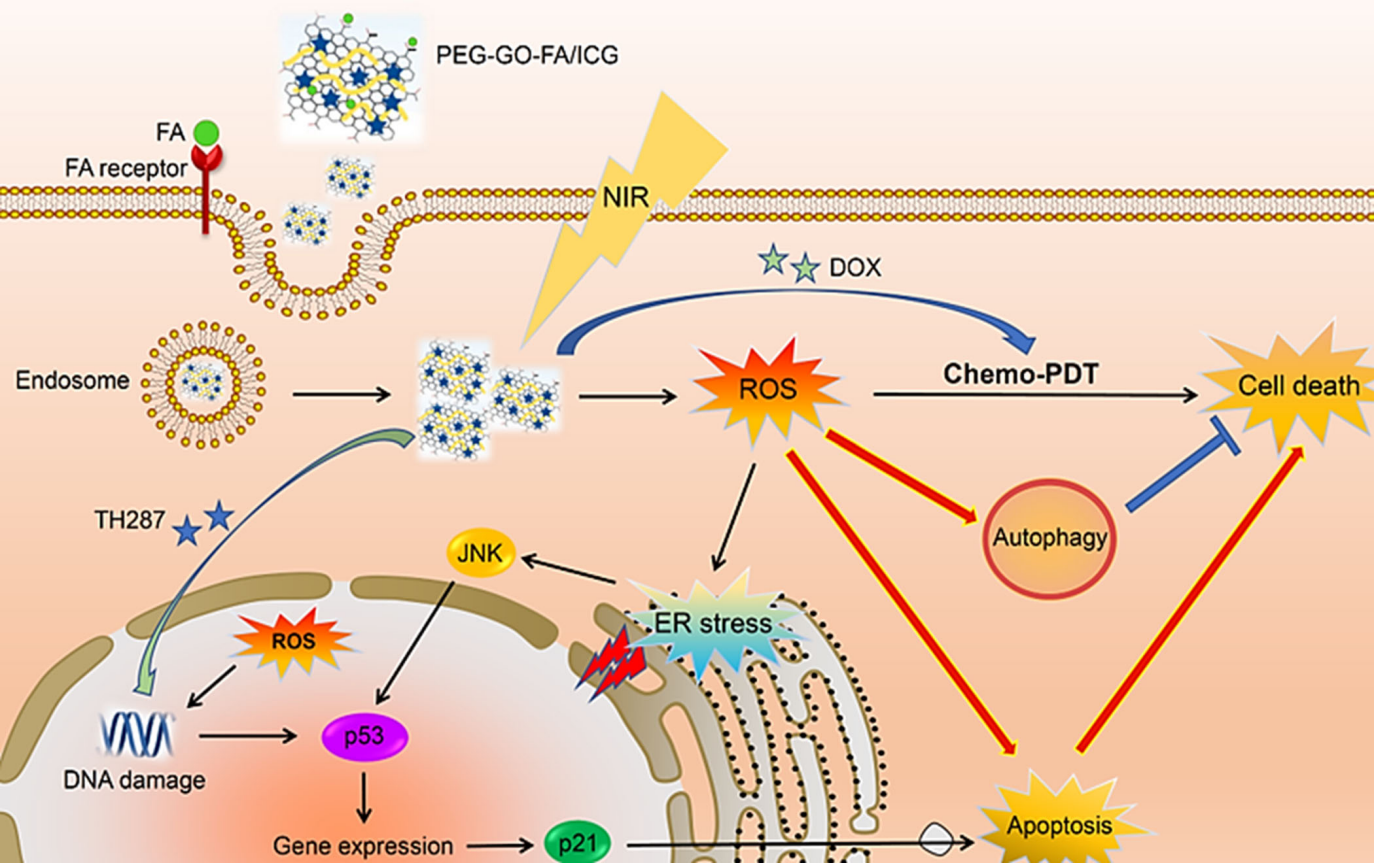
Chemo‐photodynamic cancer nanotherapy using a nanocomposite prepared from GO, polyethylene glycol (PEG), folic acid (FA), and photosensitizer indocyanine green (ICG). The nanosystem could stimulate the apoptosis and autophagy pathways through the suppression of MutT homolog 1 protein and stimulation of reactive oxygen species (ROS) accumulation; ROS helped to the endoplasmic reticulum (ER) stress‐promoted apoptosis via the JNK/p53/p21 trail. Reproduced with permission from Elsevier, 2020^109^

Liu et al.^108^ reported a drug delivery nanosystem for the synergistic chemo‐photothermal therapy of cancers using GO nanosheets; the tumor intracellular environment and photothermal heating had stimulatory effects on the release of anticancer agents from nanocarriers.^108^ On the other hand, during the non‐invasive treatment of cancers, photodynamic therapy can form significantly toxic ROS.^109^, ^110^ The suppression of MutT homolog 1 protein function (a DNA oxidative damage repair protease) can enhance the efficacy of photodynamic therapy via the improvement of cellular sensitivity to ROS. Thus, in one study, functionalized GO‐based nanosystems were prepared using PEG, folic acid, and photosensitizer indocyanine green for delivering the MTH1 inhibitor and doxorubicin. These nanosystems demonstrated chemo‐photodynamic therapy effects for inhibiting the osteosarcoma cells proliferation and migration. The improved chemo‐photodynamic therapy stimulated the apoptosis and autophagy pathways via the suppression of MutT homolog 1 protein and stimulation of ROS accumulation (Figure 5).^109^ In another study, the decoration of reduced‐GO was performed with the purpose of combinational chemo‐photodynamic cancer treatment using magnetic nanoparticles and camptothecin drug by connecting 4‐hydroxy coumarin to reduced‐GO via an allylamine linker.^109^ The nanocarriers demonstrated pH‐depended release behavior and suitable cytotoxic effects against the human breast cancer cell lines. It was revealed that free camptothecin had higher toxic effects on normal cells, and could damage the DNA. In contrast, the prepared nanosystem demonstrated good biocompatibility and no remarkable toxic effects on the normal cells (WS‐1 cells); the cell viability was ∼75% in 100 μg ml^–1^ following 24 h of the therapy. The photodynamic therapy deploying UV‐Visible irradiation (> 365 nm) could reduce the cancer cell viability to ∼38%. The laser irradiation could lead to generating higher amounts of ROS for significantly inhibition of cancerous cells; this combinational therapy strategy exhibited synergistic anti‐tumor efficiency and significant apoptosis of targeted cells.^111^

## 4 IMPORTANT CHALLENGES AND FUTURE PERSPECTIVES

Overall, the use of G‐ and GO‐based materials with elevated electrical conductivity, mechanical strength, and stiffness in the design and fabrication of anticancer nanosystems has promising advantages and unique features.^110^, ^112^ Table 1 summarizes some important examples of G‐ and GO‐based materials with their advantages and properties. However, crucial challenges regarding their cellular long‐term cytotoxicity/histopathology, immunogenicity, bio‐persistency, multi‐drug resistance, clearance mechanism, intracellular uptake, and bioaccumulation are still need to be systematically evaluated; the effects of particle size on the viability of cells have not been much examined by researchers.^22^, ^113^ Bi et al.^114^ have discussed the possibility of lung cancer metastasis/progression after long‐term pulmonary exposure of G and carbon black. Accordingly, the cell necrosis and discharge of damage‐associated molecular patterns (such as mitochondrial DNA) could have happened; the mitochondrial DNA can potently stimulate the secretion of Wnt ligands in alveolar macrophages.^114^

**TABLE 1:** G‐ and GO‐based nanosystems for cancer therapy with promising advantages

**G‐ and GO‐based nanosystems**	**Applications**	**Important features**	**Refs**.
Multifunctionalized GO	Targeted cancer therapy and drug delivery	– No noticeable toxic effects – Higher drug stacking capability – pH‐responsive drug discharge features – Particular target transport and effectual cell inhibition	^100^
Carboxymethyl cellulose‐GO	Targeted and sustained drug delivery	– No noticeable toxicity with sustained and prolonged release of doxorubicin – Incorporation of GO nanosheets highly improved the swelling capacity of hydrogels	^118^
GO	Cancer therapy and drug delivery system	– Sustained‐release nanoformulation – Improved suppression of cancer cell growth	^119^
GO‐hyaluronic acid‐Arg‐Gly‐Asp peptide	Targeted cancer therapy and anticancer drug delivery	– Low toxicity – High drug loading – Improved specificity and efficiency of anticancer drug delivery	^120^
Magnetic GO‐chitosan‐PEG‐N‐Hydroxysuccinimide	Anticancer drug delivery system	– Good biocompatibility – Low cytotoxicity – pH‐responsive controllable drug release behavior – High drug loading potentials	^121^
polyvinylpyrrolidone‐ and *β*‐cyclodextrin‐modified GO	Targeted anticancer drug delivery	– Low toxicity – pH‐dependent drug release	^122^
GO@soy phosphatidylcholine‐folic acid nanohybrid	Antitumor therapy and targeted drug delivery	– No noticeable toxicity – pH‐dependable drug release – Improved steadiness and good biocompatibility – Higher drug packing ability – Effectual cellular uptake – Regulated drug discharge	^123^
Chitosan‐grafted‐poly(methacrylic acid)/GO	Anticancer drug delivery	– No detectable toxicity – Significant biocompatibility – High drug packing capacity – pH‐dependent drug delivery performance	^124^
GO/chitosan oligosaccharide/**γ**‐polyglutamic acid	Anticancer drug delivery	– No detectable toxicity – Simple delivery and controllable anticancer drug release behavior	^125^
Superparamagnetic iron oxide‐GO	Smart nanotheranostics platform	– Good biocompatibility – pH‐dependable drug release	^126^
Chitosan‐carboxylated GO	Gene delivery	– High gene transferring properties	^127^
Modified GO	Gene delivery	– Low toxicity – Improved release of DNA – Suitable interaction with DNA and hydrophobic immune adjuvant	^128^
GO/ethylene glycol‐polycaprolactone	Anticancer drug delivery; tumor therapy	– Low cytotoxicity – Improved biocompatibility and biodegradability – High drug release and inhibition of tumor growth	^129^
GO‐nanoscale hydroxyapatite	Cancer therapy (chemotherapy and photothermal therapy)	– High biocompatibility – High photothermal therapy activity – Improved drug release behavior – High drug loading capacity	^130^
Polymer G nano‐aerogels	Anticancer drug delivery	– High anticancer drug‐releasing with pH‐dependable behavior	^131^
Starch‐G nanosheets	Anticancer drug delivery	– High anticancer drug loading capacity – Sustained‐release behavior – Good biocompatibility – Low toxicity with improved therapeutic efficacy	^132^
Reduced‐GO nanostructures	Cancer therapy and anti‐inflammatory effects	– Anti‐proliferative activity with high efficacy	^133^
Reduced‐GO nanostructures	Anticancer drug delivery	– Sustained pH‐sensitive drug release – Improved therapeutic efficacy – High drug loading capacity – High hemolytic toxicity to rabbit red blood cells	^101^
Nanoscale GO loaded with HN‐1 (a tumor‐targeted peptide)	Anticancer drug delivery	– High stability to the biological solution – High tumor‐targeting behavior – pH‐responsive drug release – High cellular uptakes and cytotoxicity toward tumor cells	^134^
D‐mannose‐mediated chitosan‐functionalized GO nanosystems	Anticancer drug delivery	– Good biocompatibility – Targeted and controlled delivery – Intracellular discharge of marine algae‐mediated anticancer drugs versus glioblastoma cancers (e.g., ulvan)	^135^
5‐Fluorouracil and curcumin loaded chitosan/reduced GO nanocomposites	Anticancer drug delivery	– Synergistic inhibitory effects against the growth of HT‐29 colon cancerous cells – Dual‐drug loading properties – Improved targeting properties	^136^

Another important issue is biological membranes that can function as barriers and restrict the diffusion of various molecules. Thus, innovative drug delivery nanosystems have to be developed with improved membrane permeability features, as it has been indicated that GO nanosheets loaded Tegafur drug had beneficial cell membrane permeability properties.^115^ Besides, the lack of enough stability in bio‐medium can hinder the cancer photothermal therapy using G‐based materials. Thus, various polymers need to be explored for the functionalization of these materials, as has been exemplified in the case of functionalized GO being modified by an amphiphilic polymer which displayed improved colloidal stability with enough cytocompatibility, suitable size distribution, and neutral surface charge.^116^ Furthermore, hybrid functional G‐based nanocomposites have been studied by researchers to improve the biocompatibility and cellular uptake features.^118^ For instance, the complex of GO and GQDs exhibited excellent photothermal effects, improved biocompatibility, and high cytotoxic performances against cancers, indicating that hybrid G‐based nanocomposites may be promising candidates for cancer theranostics and cell imaging. However, these types of hybrid nanostructures with synergistic and optimized properties need to be still further examined, and more analytical explorations are nonetheless needed for the improvement of specificity and reduction of possible toxicity.^117^

For a step toward the improvement of stability and bioactivity, natural polyphenols have been utilized in combination with the G‐based nanocomposites. Sivaperumal and co‐workers^137^ have described the preparation of silver and gold nanoparticles hybridized with reduced GO nanocomposites and deploying the anticancer flavone, chrysin with improved stability, bioactivity, and biocompatibility. These nanohybrids had enhanced cytotoxic effects against breast carcinoma cell lines with low toxicity to the normal cells; the formation of ROS discharged by the co‐existing metal ions on the reduced GO promoted apoptosis.^137^ Some natural polyphenolic flavonoids with anticancer effects (e.g., quercetin) have been utilized to generate G‐ and GO‐based nanosystems with controlled drug delivery; high cytotoxicity toward cancerous cells and targeted drug delivery properties could be attained by these nanosystems.^138^

Because of the serious adverse effects and non‐targeting disadvantages of chemotherapy tactics, researchers have instigated extensive investigations into innovative nanostructures that have led to the design of a wide range of effective nanosystems for the treatment of cancers. However, important challenges regarding the effects of particle size/morphology, chemical structures, reaction/physiological conditions, and surface chemistry on efficacy and biosafety of designed anticancer systems are crucial.^139^ Furthermore, the usage of hazardous or toxic compounds should be avoided in the process of designing these anticancer systems, preventing possible adverse health effects, skin irritations, immune reactions, and toxicity.^140^ Green and sustainable synthesis methods, as well as green functionalization processes with reproducibility and up‐scalability advantages, can be deployed for the preparation of G‐based nanosystems.^141^ Such protocols based on green chemistry for the reduction and prevention of potential environmental and health risks as well as the enhancement of biocompatibility and sustainability should be given more consideration by researchers. For instance, the fluorinated G constructed via a simple and green technique has been used as a nanocarrier for the targeted transport of curcumin to the cancerous cells with good biocompatibility in the concentration range of 100–500 μg ml^–1^; the toxic effects were dependent on the concentration of the sample.^142^ The images of confocal spectroscopy have revealed blue and red emission from the nuclei of cancerous cells because of the attendance of G‐based nanocarrier and curcumin, respectively, after 12 h of the treatment.^142^ Besides, green‐synthesized copper oxide (CuO) nanoparticles were decorated on GO nanoplatforms to perform against HCT‐116 human colon cancer cell lines (the cytotoxicity was ∼70%).^143^

**FIGURE 6:**
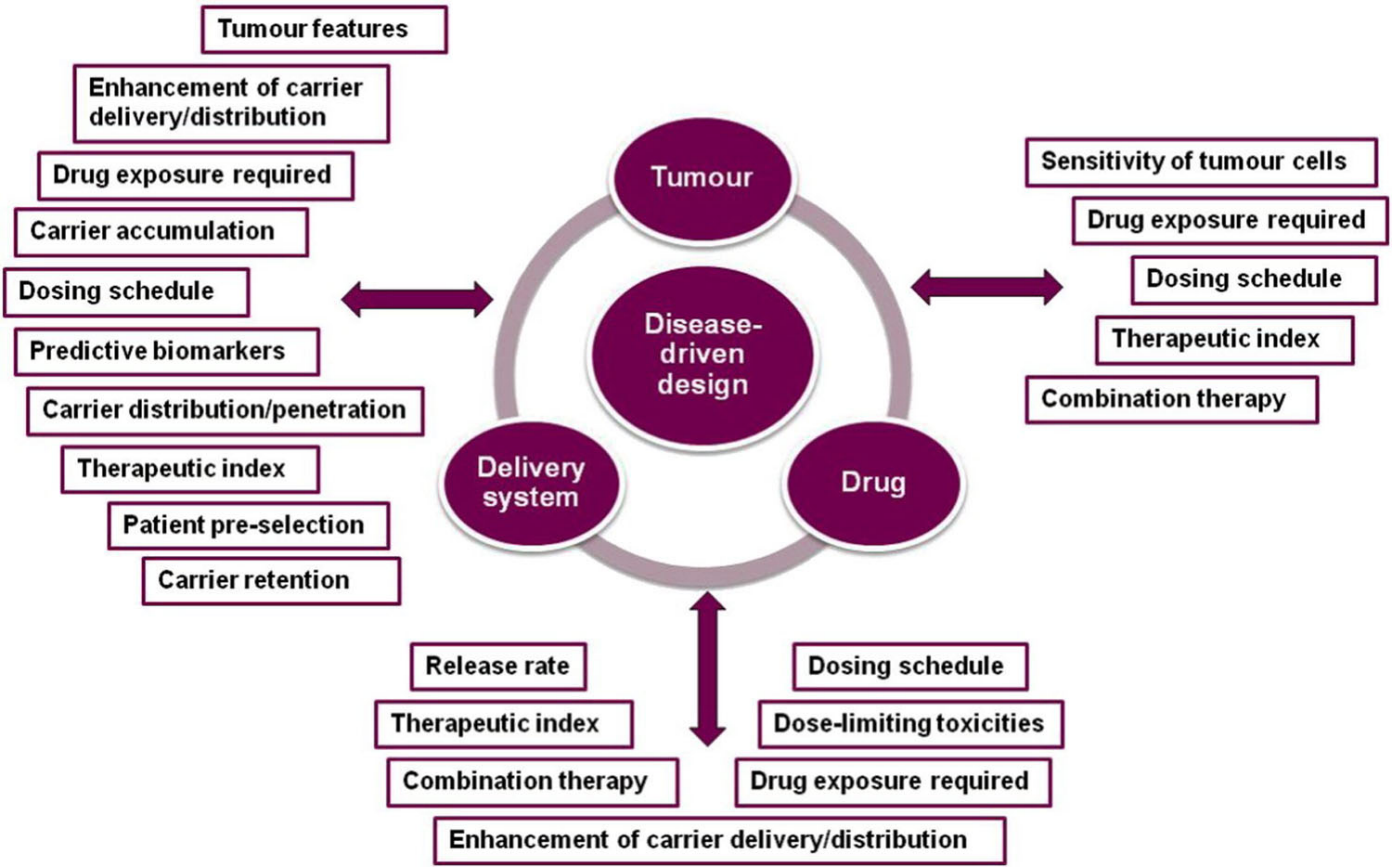
Some essential considerations for disease‐driven design and development of the nanosystem‐based delivery of antitumor or anticancer agents (therapeutics), the aim has to be on the criteria for selecting the delivery system, drug, and target patient population. Reproduced with permission from Elsevier, 2017^148^

Important criteria such as pharmacokinetics, pharmacodynamic biomarkers, and tumor responses must be assessed, especially for targeted anticancer nano‐delivery.^144^, ^145^, ^146^ For industrial manufacturing of G‐based nanomaterials with anticancer applicability, simplicity, cost‐effectiveness, and environmentally benign routes with excellent productivity are a prerequisite; both practical and theoretical studies must be specifically emphasized for the development of optimized synthesis techniques to have a seamless transition from lab‐scale to industrial production in the adaptation of conventional lab‐scale techniques.^147^ Clinical and long‐term assessments are vital after the production, which has been infrequently attended; long‐term cytotoxicity of G‐based materials and their effects on cell signaling should be clarified. Importantly, the understanding of mechanisms responsible for toxic effects can help to identify the means to reduce them, providing functionalized G‐based materials with high biocompatibility. The selection of rational criteria is of immense importance for the development of clinically successful and translatable nanomedicines. A disease‐driven strategy to develop smart drug delivery nanosystems with emphasis on significant parameters related to the drug‐delivery system and target patient population can be deployed to balance different variables to enhance the therapeutic activity (Figure 6).^148^ Furthermore, immunogenicity, inflammatory reactions, and hemocompatibility are vital criteria for anticancer employment of G‐based materials.^149^ It has been indicated that G‐based nanocomposites could have DNA or mitochondrial damage, inflammatory reactions, autophagy, necrosis, and apoptosis effects; these materials have also validated dose‐dependent toxicity behavior.^150^ In one study, hemolytic effects of GO structures were typically initiated via electrostatic interaction between these materials and red blood cell membrane, which can be circumvented by suitable surface functionalization or modification to improve the hemocompatibility.^151^ It was revealed that GO caused significant immunogenicity as confirmed by a notable upsurge of tumor necrosis factor‐α, interleukin‐6, and interleukin‐1; however, the functionalized GO structures illustrated improved immunological compatibility.^152^ For instance, interleukin‐6, interleukin‐12, tumor necrosis factor‐α, interferon γ, and monocyte chemotactic protein 1 were remarkably enhanced by applying GO structures, causing significant inflammatory effects.^153^ GO‐induced inflammatory cytokines via interaction with toll‐like receptors activated the NF‐κB trail; however, functionalized G‐based nanomaterials could evade such inflammatory effects by macrophages through weakening the opsonin‐protein interaction.^151^

Given the widespread use of G‐based materials especially in biomedicine, precise deliberation of their toxicity is critical.^154^ There are numerous studies showing the dose‐dependent toxicity of G‐based materials to animals and human cells, including lung granuloma generation, injury of liver/kidney, reduced viability of cells, and apoptosis. Some important parameters such as the functionalization, surface structure, aggregations, lateral size, corona effect, charge, and impurities have effects on the toxicological profile of these materials. On the other hand, distinct consideration should be exercised to study the possible events and mechanisms related to the toxicity of G‐ and GO‐centered entities such as apoptosis, DNA damage, oxidative stress, necrosis, physical destruction, inflammatory reactions, and autophagy.^154^

## 5 CONCLUSION AND FUTURE OUTLOOKS

In conclusion, G‐ and GO‐based materials are of special interest in cancer therapy and have been widely studied in the past few decades. These structures have been studied for various pharmaceutical and biomedical appliances owing to their unique physicochemical properties such as two‐dimensional planar structures, large surface areas, high chemical/mechanical stability, and significant conductivity. However, pristine G and GO may suffer from unfavorable surface chemistry and low biocompatibility, thus various covalent or non‐covalent functionalization tactics have been deployed to improve their properties. Important themes associated with the sustained release of anticancer drugs as well as mechanistic insights of anticancer agents’ discharge from G‐based nanoplatforms still await comprehensive study. Likewise, the efficient accumulation of anticancer drugs in tumor targets/tissues, controlled cellular uptake properties, tumor‐targeted drug release behavior, and selective toxicity toward the cells are crucial criteria that need to be met for developing future anticancer G‐based nanosystems. Overall, functionalized G‐based nanosystems with better biodistribution of drugs, low adverse effects on healthy cells, high selectivity/sensitivity, and higher local therapeutic absorption have garnered a lot of attention. For biomedical and clinical applications of G‐based materials, precise deliberation of their biosafety and toxicity issues is critical.

## FUNDING INFORMATION

Not applicable.

## CONFLICT OF INTEREST

Dr. Rajender S. Varma is on the editorial board of the journal, *MedComm*. The other authors declare no conflict of interest and no financial support toward this research, and/or for the authorship and publication of this article.

## ETHICS APPROVAL

Not applicable.

## AUTHOR CONTRIBUTIONS

All authors including A.S., S.I., and R.S.V., have contributed equally to conceptualize, review the papers, designing the discussion, writing the initial draft, and preparing the final document.

## Data Availability

Not applicable.
